# Psychosocial Status of Liver Transplant Candidates in Iran and Its Correlation with Health-Related Quality of Life and Depression and Anxiety

**DOI:** 10.1155/2015/329615

**Published:** 2015-11-15

**Authors:** Maryam Banihashemi, Mohsen Hafezi, Mohsen Nasiri-Toosi, Ali Jafarian, Mohammad Reza Abbasi, Mohammad Arbabi, Maryam Abdi, Mahzad Khavarian, Ali-Akbar Nejatisafa

**Affiliations:** ^1^Psychosomatic Research Center, Tehran University of Medical Sciences, Tehran 1419733141, Iran; ^2^Liver Transplantation Research Center, Tehran University of Medical Sciences, Tehran 1419733141, Iran; ^3^Nephrology Research Center, Tehran University of Medical Sciences, Tehran 1419733141, Iran; ^4^Psychiatry and Psychology Research Center, Tehran University of Medical Sciences, Tehran 1333715914, Iran; ^5^Department of Psychiatry, Psychosomatic Research Center, Tehran University of Medical Sciences, Tehran 1419733141, Iran

## Abstract

*Objectives*. The study was aimed at providing a psychosocial profile for Iranian liver transplant candidates referred to an established liver transplantation program.* Material and Methods*. Patients assessed for liver transplant candidacy in Imam Khomeini Hospital (Tehran, Iran) between March 2013 and September 2014 were included. The following battery of tests were administered: Psychosocial Assessment of Candidates for Transplant (PACT), the Short-Form health survey (SF-36), and Hospital Anxiety and Depression Scale (HADS).* Results*. Psychosocial assessment in 205 liver transplant candidates revealed significant impairments in several SF-36 domains; social functioning was the least and physical functioning was the most impaired domains. The prevalence of cases with probable anxiety and depressive disorders, according to HADS, was 13.8% and 5.6%, respectively. According to PACT, 24.3% of the assessed individuals were considered good or excellent candidates. In 11.2%, transplantation seemed poor candidate due to at least one major psychosocial or lifestyle risk factor. Poor candidate quality was associated with impaired health-related quality of life and higher scores on anxiety and depression scales (*p* < 0.05).* Conclusions*. Transplant programs could implement specific intervention programs based on normative databases to address the psychosocial issues in patients in order to improve patient care, quality of life, and transplant outcomes.

## 1. Introduction

In 2001, End Stage Liver Failure (ESLF) accounted for about 800,000 deaths worldwide [1]. The 5-year mortality rate of ESLF is estimated to be 50% [2]. Liver transplantation is currently the only available treatment that can reverse, albeit temporarily, the deteriorating trajectory of events leading to death, thereby significantly increasing patient's life-expectancy [1].

The psychiatrist plays a key role in the transplant team; the services offered by the psychiatrist span from the initial stages of the assessment process to the indefinite added life years gained by the patient after the transplant [3]. In the pretransplant period, the psychiatrist evaluates the patient and forms his/her expert opinion regarding the presence of absolute contraindications to receiving an organ [4, 5]. Preoperatively, adjustment disorders, major depression, anxiety disorders, and other Axis I disorders are highly prevalent in transplant candidates. Current substance dependence, previous deliberate self-harm, and severe mental disorders are among the contraindications of the organ transplantation [4]. If psychiatric disorders are left untreated prior to transplantation, they can harbor negative implications for the procedure's outcome including refusal to accept transplantation and poor self-care and nonadherence to treatments [6]. Moreover, the patient's compliance to the ineluctable lifestyle change demanded by the transplant and the long-term treatment with immunosuppressive medications is also negatively influenced by psychopathology before and after the procedure [7, 8]. Lack of adherence to medications could in turn translate into more rehospitalization episodes, increased risk of organ rejection, and subsequently shorter posttransplant survival [9, 10].

The demand for donor livers by far exceeds its supply and a sizable proportion of patients with ESLF decease while waiting for a donor organ. In this situation, the principal role of the psychiatrist appears to be helping the majority of transplant candidates who never make it to transplant, to cope with the disease. These patients need help to adjust with the debilitating nature of the chronic disease, to get in touch with the reality of not ever getting a transplant, to become prepared for the lengthy waiting time, and to overcome fears, distresses, and disabilities that diminish their quality of life [3, 11, 12].

Interventions planned to address psychological distress and improve quality of life in the population of patients awaiting liver transplant are not effective unless normative situation analyses regarding baseline status of the patients are available [13]. Several pretransplant psychiatric assessments of liver transplant candidates have been published so far [12–18]. Yet, to our knowledge, no reports to date have been available from Iran. In Iran, the first liver transplant was conducted in 1993 in Shiraz [19]. In June 2000, the parliament ratified the Organ Transplantation Act which helped expand organ transplantation programs across the country [20]. Shortly after, in 2001, a liver transplantation program was developed at Imam Khomeini Hospital (Tehran, Iran) as the second liver transplantation center in the country. The present study was conducted to provide a preliminary normative database of the Iranian liver transplant candidates referred for assessment in Imam Khomeini Hospital.

## 2. Materials and Methods

### 2.1. Patients

In the present study, all patients assessed for liver transplant candidacy in Imam Khomeini Hospital (a large referral center associated with Tehran University of Medical Sciences, Tehran, Iran) in a period of 18 months, between March 2012 and September 2013, were included. Demographic characteristics of patients including age, gender, education, marital status, and occupation were recorded using a standard predesigned questionnaire. Model for End-Stage Liver Disease (MELD) [21] scores were also retrieved from patients' files. The following battery of tests were administered. As part of their pretransplant psychiatric assessment, a psychiatrist (NS) interviewed the patients and completed the Psychosocial Assessment of Candidates for Transplant (PACT) screening tool. The Short-Form health survey (SF-36) was administered to assess the health-related quality of life (HrQoL) in patients. Moreover, Hospital Anxiety and Depression Scale (HADS) was used to determine the levels of anxiety and stress in pretransplant patients. At the time of interview, no patients were diagnosed with delirium, hepatic encephalopathy, or cognitive impairment significant enough to render the assessments unreliable. Written informed consent for use of the collected data in research, granting that the confidentiality of the evaluations is not breeched, was obtained from all patients prior to enrollment. All interviews and assessments were carried out in compliance with the principles of good clinical practice delineated by the latest revision of Declaration of Helsinki. The Ethics Committee of the Tehran University of Medical Sciences also approved the protocol.

### 2.2. Questionnaires

#### 2.2.1. SF-36

SF-36 is a widely used, valid, and reliable measure of HrQoL that has been translated into numerous languages including Persian. The 36-item, self-assessment questionnaire evaluates eight domains of HrQoL which are physical functioning (10 items), physical role limitation (4 items), bodily pain (2 items), general health (5 items), vitality (4 items), social functioning (2 items), emotional role limitation (3 items), and mental health (5 items) [22]. The response to the items can be yes/no, 3-, 5-, or 6-point Likert-type scale. In the present study, the Persian translation of the questionnaire was used [23]. In the Persian version, Cronbach's *α* for individual domains was either good or excellent (ranging from 0.77 to 0.90), except for the vitality domain for which the level of internal consistency was acceptable (Cronbach's *α* = 0.65) [23].

#### 2.2.2. HADS

HADS is a self-administered, 14-item instrument for detecting anxiety and depression in inpatient as well as outpatient settings [24]. HADS has two subscales; of the 14 items, seven deals with symptoms of anxiety (anxiety subscale; HADS-A) and the other seven probe depressive symptoms (depression subscale; HADS-D). Each item is answered on a 4-point Likert-type scale (0–3). On each subscale, the score can range between 0 and 21. Patients scoring 0–7 are considered noncase, those with scores 8–10 are labeled borderline, and scores 11 and above indicate “caseness” or clinically significant levels of anxiety/depression [24]. In the present study, the translated Persian version of the questionnaire was used [25]. Cronbach's *α* for the Persian version has been reported to be 0.78 and 0.86 for anxiety and depression subscales, respectively [25].

#### 2.2.3. PACT

PACT is a 10-item, interviewer-administered tool developed for the screening of psychosocial risk factors in organ transplant candidates. Items are decorated in four distinct categories of (I) social support, (II) psychological health, (III) lifestyle factors, and (IV) understanding of the process of transplant and follow-up [4]. The interviewing psychiatrist subjectively evaluates each section and scores the individual items on a 5-point Likert-type scale with higher scores indicating better eligibility for receiving organ transplant. At the end of the inventory, the interviewer is asked to provide a final rating of candidate quality, based on his overall judgment of the patient and not only by averaging individual categories. Despite its subjective nature, it has been shown that PACT has an excellent interrater reliability (intraclass correlation = 0.85) [4].

### 2.3. Statistical Analysis

For statistical analyses, the Statistical Package for Social Sciences (SPSS) version 19 for Windows (IBM Corp., New York, USA) was used. Continuous variables are presented as mean (standard deviation: SD) and categorical variables as proportions. For the SF-36 and HADS scores, in addition to mean (SD), range (minimum–maximum) and median (interquartile range) are also presented. Mean scores of SF-36 domains and also HADS were compared across populations using Welch's unpaired* t*-test which does not assume the between-populations equality of variance.

SF-36 and HADS scores were compared across PACT overall candidate quality categories using analysis of variance (ANOVA). Since the fifth category (excellent candidate) had only two members, it was pooled with the fourth one (good candidate). In all tests, a *p* value of less than 0.05 was considered necessary to reject the null hypothesis.

## 3. Results

Completed assessments and questionnaires were available for 205 liver transplant candidates. Baseline characteristics of evaluated patients are presented in Table 1. The minimum and maximum ages of the participants were 15 and 67 years, in order. Men comprised three-fifths of the participants. About one-third of the evaluated subjects were either unemployed or retired or had to leave work due to disability. The average MELD score was 16.4, ranging between 4 and 30.

Mean, SD, range, minimum, maximum, median, and interquartile range of the SF-36 domains, and also HADS-A and HADS-D are shown in Table 2. Among SF-36 domains, social functioning was the least impaired, whereas subjects showed the highest level of impairment in physical role functioning. The mean scores for HADS-A and HADS-D were 5.8 and 5.2, in order. The proportion of noncase, borderline, and case for anxiety disorders were 70.9%, 15.3%, and 13.8%, respectively. For the depression subscale, the proportion of noncase was 76.6%; 17.8% were diagnosed as borderline, and only 5.6% had scores above the level of clinical significance.

**Figure 1:**
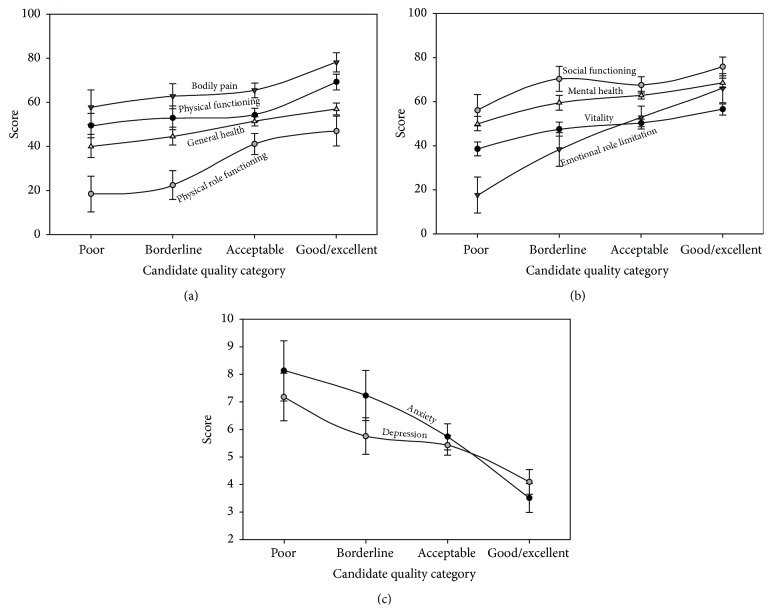
*Association between liver transplant candidate quality and Health-related quality of life (a, b) and between liver transplant candidate quality and anxiety and depression (c)*. (a, b) Scores in SF-36 domains linearly increase when moving from poor to good/excellent candidate quality categories. Physical functioning (*F* = 7.287, *p* = 0.008), physical role functioning (*F* = 8.663, *p* = 0.004), bodily pain (*F* = 6.042, *p* = 0.015), general health (*F* = 11.478, *p* = 0.001), vitality (*F* = 17.616, *p* < 0.001), social functioning (*F* = 4.455, *p* = 0.036), emotional role functioning (*F* = 18.148, *p* < 0.001), and mental health (*F* = 19.341, *p* < 0.001). (c) HADS anxiety and depression scores linearly decrease when moving from poor to good/excellent candidate quality categories. Anxiety (*F* = 17.526, *p* < 0.001), depression (*F* = 11.673, *p* = 0.001). Abbreviations: SF-36, Short-Form health survey; HADS, Hospital Anxiety and Depression Scale.

The results of the PACT are presented in Table 3. Only one-fourth of the assessed individuals were considered good or excellent candidates. In 19 patients (11.2%), the overall assessment of the psychiatrist was that the transplant is contraindicated due to some major impairment in psychological, social, or lifestyle risk factor. SF-36 and also HADS scores were compared across PACT overall candidate quality categories and the findings are presented in Figure 1. As demonstrated, in all eight SF-36 domains, significant linear trends between candidate quality and less impairment in HrQoL were observed (Figures 1(a) and 1(b)). When moving across candidate quality categories, HADS-A and HADS-D scores linearly decreased (Figure 1(c)).

## 4. Discussion

In the present study, the HrQoL and also the levels of anxiety and depression based on pretransplant psychiatric assessment of liver transplant candidates were described. Moreover, the findings from PACT conducted by a psychiatrist were presented. Montazeri and colleagues validated and administered the SF-36 questionnaire on a community sample of 4163 adults aged 15 and older. In all eight HrQoL domains, scores of our liver transplant candidates were lower than those of the general population of Iran and the largest difference was found for physical role functioning [23]. The physical functioning of patients with severe liver cirrhosis is considerably limited by major complications like chronic encephalopathy, ascites, poor nutrition, and fatigue [13]. This impairment in physical domain could play a synergistic role for impairment in social and psychological domain. HrQoL assessments for liver transplant candidates in other countries have also been published [13, 26]. When compared with the liver transplant candidates of the Florida cohort (1991–1996), it was found that the levels of HrQoL for Iranian patients are generally higher in all the domains examined. Although, in both samples, the lowest score belonged to the physical role functioning limitation, the average score of this domain in the Iranian patients was more than twice the figure observed in the Florida cohort. In addition to the distinct time frame of two studies, the difference of HrQoL in them could be ascribed to dissimilarities between cultures as well as the age and MELD score differences between the two populations.

Transplant candidates experience a wide range of psychosocial stressors before the transplant [27]; available studies have identified a large proportion of these patients to be afflicted by clinically significant psychiatric disorders [13, 18, 28]. Patients fear the possibility of an impending death and might be frustrated that they cannot change the trajectory of their life [15]. If they are wait-listed for the transplant, the stress of not knowing when they might get a call might be debilitating [11, 17, 27]. Indeed, they are often asked to prepare themselves for two completely different scenarios; getting a transplant and handling the strenuous process of liver transplant or coming to accept the fact that their condition is rapidly deteriorating and transplant might not be an option for them [27]. They also have to adjust themselves with the disabilities and losses of functioning imposed by the chronic disease itself and also cope with the ramifications in areas of work, family, and social life [12, 17, 29]. Last but not least, the pretransplant evaluation process itself could also put the patient under copious amounts of stress [15].

The frequency of anxiety and depression significant enough for clinical intervention was detected in 13.8% and 5.6% of the patients, respectively. In the Florida cohort study, 39% of the patients exhibited anxiety symptoms in the clinical range and 18% had moderate or severe depression [13]. Similarly, in a study of 165 liver transplant candidates in Italy, the prevalence of anxiety in the clinical range was reported to be 55% [28]. Ten percent of patients had also depressive disorders based on the diagnostic and statistical manual of mental disorders (fourth edition) criteria [28]. On the other hand, as measured by the HADS questionnaire, mean scores for anxiety and depression were comparable to those of liver transplant patients in Switzerland [11] or Brazil [30].

In the present study, the PACT screening tool showed that the majority of interviewed candidates had no identifiable contraindication for receiving transplant. The most prevalent predictor of poor outcome was in the category of alcohol or drug abuse in which six patients were considered to be actively using or were reluctant to abstain. Other important findings were relatively low scores of the participants in knowledge and life style category of PACT. Only 9 patients (5.3%) and 3 patients (1.8%) had the highest scores for healthy lifestyle and knowledge, respectively. As these two factors have an important role in subsequent adherence to treatment, the transplant team should provide an educational program for increasing knowledge and promoting healthy life style in the patients.

PACT assessments in our study were conducted by a single psychiatrist in charge of the psychological assessments in the transplant team. Although previous experience with the PACT has shown there is excellent interrater agreement between interviewers [4], we further limited the presence of this bias by taking advantage of a single observer.

In our sample of Iranian pretransplant patients, better PACT score is significantly associated with better HrQoL and also less anxiety and depression. Based on these observations, it can be concluded that PACT, despite its brief and subjective nature, provides valuable information regarding the presence of psychopathology in patients and therefore clinical decisions regarding the quality of a candidate from a psychological standpoint could be based on PACT scores. Moreover, we found herein that patients who are considered poor transplant candidates are also the ones with the lowest HrQoL and the highest level of anxiety and depression. This presents an important challenge for the psychiatrist and highlights the need for evidence-based intervention plans specifically tailored to increase the HrQoL and decrease psychopathology in the subset of patients with ESLF who are not on the transplant short-list [11, 14].

A number of limitations in the present study deserve mention. We acknowledge that the presented psychiatric assessment of candidates is by no means comprehensive. In order for the psychiatric assessment normative database to be used in predictive models of patient outcomes and survival after transplant, contributing medical factors including the etiology of the liver disease, disease severity, frequency and severity of hepatic encephalopathy, gastroesophageal bleeding, ascites, and spontaneous bacterial peritonitis and also other medical comorbidities not directly related to the chronic liver disease should be evaluated and be used as the basis of decision-making. A number of studies have linked the severity of liver disease as the most important or at least have identified it as a major determining factor in the fate of the transplant [28, 31]. Therefore, a joint framework that incorporates both medical and psychiatric assessments of a likely candidate needed for meaningful inferences regarding the prognosis of the disease could be drawn.

Transplant programs should develop and implement specific evidence-based intervention programs, derived from their normative databases to address the psychosocial issues in patients undergoing transplantation as well as the great majority that never find a donor. According to the finding of this study, interventions should be vectored toward the following aspects: helping patients to cope with their chronic disease, increasing the knowledge of the patients about transplantation and healthy life style, treatment of major psychiatric disorders, and providing more social and economic support for patients in order to improve their quality of life.

## Figures and Tables

**Table 1:** Baseline characteristics of Iranian liver transplant candidates (*n* = 205).

Variable	Statistics
Age, mean (SD)	44.3 (13.1)
Sex (%)	
Female	79 (38.5%)
Male	126 (61.5%)
Marital status (%)	
Single	41 (20.0%)
Married	156 (76.1%)
Divorced/widowed	8 (3.9%)
Job status (%)	
Paid work	99 (48.3%)
Homemaker	31 (15.1%)
Student	3 (1.5%)
Disabled/unemployed/retired	72 (35.1%)
Highest level of education completed (%)	
Illiterate	22 (10.7)
Elementary school	16 (7.8)
< high school diploma	76 (37.1)
High school diploma or above	91 (44.4)
MELD score, mean (SD)	16.4 ± 4.7

MELD, Model for End-Stage Liver Disease.

**Table 2:** Health-related quality of life, anxiety, and depression among Iranian liver transplant candidates (*n* = 205).

Variable	Mean (SD)	range (minimum–maximum)	Median (interquartile range)
SF-36			
Physical functioning	58.0 (27.1)	100.0 (0.0–100.0)	60.0 (40.0–80.0)
Physical role functioning	40.2 (42.9)	100.0 (0.0–100.0)	25.0 (0.0–100.0)
Bodily pain	67.4 (30.1)	100.0 (0.0–100.0)	67.5 (45.0–100.0)
General health	51.2 (20.1)	90.0 (10.0–100.0)	50.0 (35.0–67.5)
Vitality	50.2 (16.0)	80.0 (10.0–90.0)	50.0 (40.0–60.0)
Social functioning	70.0 (30.2)	100.0 (0.0–100.0)	75.0 (50.0–100.0)
Emotional role functioning	50.6 (45.2)	100.0 (0.0–100.0)	66.7 (0.0–100.0)
Mental health	62.0 (16.3)	80.0 (12.0–92.0)	64.0 (52.0–76.0)
HADS			
HADS-anxiety	5.8 (4.2)	20.0 (0.0–20.0)	5.0 (3.0–8.0)
HADS-depression	5.2 (3.3)	17.0 (0.0–17.0)	5.0 (3.0–7.0)

SF-36, Short-Form health survey; HADS, Hospital Anxiety and Depression Scale.

**Table 3:** Psychosocial Assessment of Candidates for Transplantation (PACT) Scores among Iranian liver transplant candidates (*n* = 205).

PACT Items	Scores				
	1	2	3	4	5
	*n* (%)				
(I) *Social support*					
Family or support system stability	0 (0.0)	20 (11.8)	45 (26.6)	89 (52.7)	15 (8.9)
Family of support system availability	0 (0.0)	24 (14.2)	56 (33.1)	75 (44.4)	14 (8.3)

(II) *Psychological health*					
Psychopathology, stable personality factors	1 (0.6)	16 (9.5)	55 (32.5)	80 (47.3)	17 (10.1)
Risk for psychopathology	1 (0.6)	39 (23.1)	71 (42.0)	45 (26.6)	13 (7.7)

(III) *Lifestyle factors*					
Healthy lifestyle, ability to sustain change in lifestyle	1 (0.6)	17 (10.1)	85 (50.3)	57 (33.7)	9 (5.3)
Drug and alcohol use	6 (3.6)	5 (3.0)	6 (3.6)	18 (10.7)	134 (79.3)
Compliance with medications and medical advice	0 (0.0)	27 (16.0)	65 (38.5)	67 (39.6)	10 (5.9)

(IV) *Understanding of the processes of transplant and follow-up*					
Knowledge and education	3 (1.8)	61 (36.1)	82 (48.5)	20 (11.8)	3 (1.8)

*Overall impression of candidate quality*	0	1	2	3	4
	19 (11.2)	28 (16.6)	81 (47.9)	39 (23.1)	2 (1.2)
